# Temperature sensing based on slow light via stimulated Brillouin scattering in M-shaped few-mode fiber

**DOI:** 10.1371/journal.pone.0342177

**Published:** 2026-02-13

**Authors:** Li-Jun Li, Shang-Lin Hou

**Affiliations:** 1 School of Electronic Information and Electrical Engineering, Shangluo University, Shangluo, Shaanxi, China; 2 Institute of Quantum Optics and Quantum Information, Shangluo University, Shangluo, Shaanxi, China; 3 School of Science, Lanzhou University of Technology, Lanzhou, Gansu, China; Manipal Academy of Higher Education, INDIA

## Abstract

This study investigates temperature sensing based on slow light generation via stimulated Brillouin scattering in an M-shaped few-mode fiber. The temperature-dependent properties of four optical modes (LP_01_, LP_11_, LP_21_, and LP_02_) over a range from –20 °C to 80 °C are analyzed using the full-vectorial finite element method. Results show that the effective refractive index and Brillouin frequency shift of all modes increase with temperature, while the effective mode areas of all modes decrease. The LP_02_ mode exhibits exceptional temperature sensitivity, showing a significant rise in slow light time delay from 0 ns to 514.9 ns, along with a growth in the pulse broadening factor. In contrast, the LP_01_ mode maintains the largest time delay across the temperature range but shows less temperature-induced variation. The results demonstrate a trade-off between slow light enhancement (time delay) and signal distortion (pulse broadening). This study confirms that the LP_02_ mode in the M-shaped few-mode fiber is highly suitable for developing sensitive fiber-optic temperature sensors, providing a theoretical foundation for optimizing slow light applications in sensing.

## 1. Introduction

Driven by breakneck AI progress and an explosion in computing needs—including soaring demand for massive-scale computing clusters—the backbone network, as the foundational communications infrastructure, demands a significant expansion in bandwidth capacity [1–3]. Space Division Multiplexing (SDM), a pivotal innovation, transcends the limitations of traditional optical fibers, offering a potent solution for substantially boosting the capacity of transmission systems [4–6]. Among the various SDM approaches, M-shaped few-mode fibers (M-FMFs) have attracted significant research interest due to their potential for high-capacity, weakly-coupled mode-division multiplexing transmission, which positions them as a key focus in next-generation optical communication research.

Slow light is a physical phenomenon in which the group velocity of light is significantly reduced as it propagates through specific media [7,8]. This effect has found important applications in fields such as optical communication and optical fiber sensing. Several methods have been developed to generate slow light, including optical parametric amplification (OPA) [9], stimulated Brillouin scattering (SBS) [10], and stimulated Raman scattering (SRS) [11]. Among these, SBS has proven to be a particularly effective technique, owing to advantages such as a widely tunable wavelength range, relatively simple system requirements, and excellent compatibility with standard communication systems [12].

Stimulated Brillouin scattering is one of the major nonlinear effects induced by the interaction between light waves and acoustic waves [13]. When light propagates through a fiber, acoustic waves are excited by the electrostriction force induced by pump light and a fraction of the acoustic-waves-backscattered light which undergoes a frequency downshift (Stokes) or upshift (anti-Stokes). While extensively studied in single-mode fibers (SMFs), research on SBS-based slow light in few-mode fibers (FMFs) is comparatively less developed [14,15]. In FMFs, the Brillouin gain spectrum (BGS) reveals greater complexity due to multimodal interactions, wherein intramodal SBS typically dominates over intermodal processes [16]. This unique characteristic has spurred significant interest in utilizing FMFs for advanced Brillouin sensing applications, such as multi-parameter detection [17–20] and high-precision temperature measurement [21–23]. However, most prior work has focused on experimental BGS characterization or sensor demonstration. A systematic and parametric understanding of how temperature influences fundamental fiber properties—such as the effective mode area and modal refractive indices—within a specific FMF geometry remains elusive. Consequently, the quantitative relationship between these temperature-dependent parameters and key slow light performance metrics, including time delay and Brillouin threshold, remains largely unexplored. This knowledge gap consequently obstructs the rational design and performance optimization of FMF-based slow-light devices and sensors.

To bridge this knowledge gap, this work presents a foundational and comparative theoretical investigation into the temperature-dependent parametric properties of an M-FMF for SBS slow light generation. We develop an analytical model to quantify how variations in temperature affect key fiber parameters, such as the effective refractive index and effective mode area, and how these changes consequently govern the slow light performance for the supported LP_01_, LP_11_, LP_21_, and LP_02_ modes. The core objective is to establish a detailed parametric framework that elucidates the underlying physical dependencies specific to the M-FMF geometry. The findings aim to provide critical insights and a solid theoretical foundation for the future design and performance optimization of M-FMF-based slow light devices and high-sensitivity temperature sensors.

## 2. Theoretical analysis

Suppose the pump light is copolarized with the signal light, the three-wave coupled equations in the M-FMF are indicated as follows [24,25] (Critical parameters, including the Brillouin linewidth and acoustic damping, are treated as fixed; pump depletion is neglected. These simplifications, while necessary for this comparative study, mean the presented time delays represent intrinsic upper bounds under ideal gain conditions. Quantitative system-level predictions will require future models that incorporate these dynamic parameters).


$$\left\{ \begin{array}{c} @l@-\frac{\partial E_{p}}{\partial z}+\frac{n_{fg}}{c}\frac{\partial E_{p}}{\partial t}=-\frac{\alpha }{\text{2}}E_{p}+ig_{\text{2}}E_{s}Q\mathrm{\backslash vspace}1.5mm\\ \frac{\partial E_{s}}{\partial \text{z}}+\frac{n_{fg}}{c}\frac{\partial E_{s}}{\partial t}=-\frac{\alpha }{\text{2}}E_{s}+ig_{\text{2}}E_{p}Q^{*}\mathrm{\backslash vspace}1.5mm\\ \frac{\partial Q}{\partial t}+\left(\frac{\Gamma_{B}}{\text{2}}-i\Delta \omega \right)Q=i\frac{g_{\text{1}}}{\eta }E_{p}E_{s}^{*} \end{array}\right.,$$ (1)

where $E_{p}$, $E_{s}$, and Q are described as [26,27] $E_{p}=\sum_{i}E_{p,i}$, $E_{s}=\sum_{j}E_{s,j}$, and $Q=\sum_{k}Q_{k}$, here $E_{p,i}$, $E_{s,j}$, and $Q_{k}$ are the amplitudes of the *i*-th pump wave, the *j*-th Stokes wave and the *k*-th acoustic wave, respectively. $n_{fg}$ is the group index without SBS, c is light velocity in vacuum, α is loss coefficient of fiber, $\Gamma_{B}/2\pi$ is the full-width at half maximum bandwidth (FWHM) of Brillouin resonance, $\Delta \omega \text{=}\omega_{p}-\omega_{s}-2\pi f_{B}$ is the detuning of angular frequency, $g_{\text{1}}$ and $g_{\text{2}}$ are the coupling coefficients of pump-Stokes and acousto-optic wave, separately.

The acoustic field is treated within an effective medium approximation. A detailed analysis of acoustic mode confinement and its temperature-dependent loss, while important for exact quantitative predictions, is beyond the scope of this comparative study focused on relative optical-mode sensitivity. The Brillouin frequency shift (BFS) $f_{B,ijk}$ of the *k*-th acoustic mode for an optical mode pair is expressed as [27]


$$f_{B,ijk}=\frac{\nu_{eff,k}}{\lambda_{p}}(n_{eff,i}+n_{eff,j}),$$ (2)

Where $n_{eff,i}$ is the effective index of the *i*-th optical mode, $\nu_{eff,k}=\omega_{a,k}/\beta_{a,k}$ is the velocity, $\beta_{a,k}$ is propagation constant of the *k*-th acoustic wave.

The SBS effect happens when the input power is higher than Brillouin threshold, which is represented as [28]


$$P_{th,ij}=\frac{21A_{eff,ij}}{g_{0,ij}L_{eff}},$$ (3)

where $L_{eff}=\left(1-e^{\left(\alpha L\right)}\right)/\alpha$ is the effective length of M-FMF, L is the real length, $A_{eff,ij}$ is the effective mode area in which the SBS effect occurs, and it is given by [29]


$$A_{eff,ij}=\frac{\left({\iint \left|E_{p,i}\left(x,y\right)\right|}^{\text{2}}dxdy\right)\left({\iint \left|E_{s,j}\left(x,y\right)\right|}^{\text{2}}dxdy\right)}{\iint {\left|E_{p,i}\left(x,y\right)\right|}^{\text{2}}{\left|E_{s,j}\left(x,y\right)\right|}^{\text{2}}dxdy},$$ (4)

Here $E_{s,j}\left(x,y\right)$ and $E_{p,i}\left(x,y\right)$ are the electric-field distributions of the *j*-th Stokes and *i*-th pump wave, separately.

The difference between the transmitting time of a pulse without and with SBS is defined as time delay, which is taken the form [24]


$$\Delta T_{d,ij}=\frac{G_{ij}}{\Gamma_{B}},$$ (5)

where $G_{ij}=g_{0,ij}P_{p}L_{eff}/A_{eff,ij}$ is Brillouin gain, $P_{p}$ is the pump power, $g_{0,ij}$ is Brillouin gain coefficient, ${\text{g}}_{0,ijk}=I_{k}\times 4\pi n_{eff,i}^{\text{8}}p_{12}^{\text{2}}/(c\lambda_{p}^{\text{3}}\rho_{\text{0}}f_{B,ijk}\Delta f_{B})$, here $\rho_{\text{0}}\text{=}2203kg/m^{\text{3}}$, $\Delta f_{B}$ is FWHM which is assumed to be 35MHz [27], $p_{12}=0.271$ is the photo-elastic coefficient, $I_{k}$ represents the overlap integral and is given by $I_{k}={\left({\iint \left|E\right|}^{\text{2}}u_{k}^{*}dxdy\right)}^{\text{2}}/\left({\iint \left|E\right|}^{\text{4}}dxdy{\iint \left|u_{k}\right|}^{\text{2}}dxdy\right)$. The modal distributions of both optical mode and acoustic mode determine the value of overlap integral which reflects the SBS efficiency.

The pulse broadening factor is expressed as [24]


$$B_{ij}=\frac{\tau_{out}}{\tau_{in}}={\left[1+\frac{16\left(ln2\right)}{\tau_{in}^{\text{2}}\Gamma_{B}^{\text{2}}}G_{ij}\right]}^{1/2},$$ (6)

$\tau_{in}$ and $\tau_{out}$ denote the pulse width of input and output Gaussian signals, respectively. Eq. (6) indicates that pulse broadening factor is greater than unity.

In the simulation, the variations of the refractive index and acoustic velocity of M-FMF with the temperature are indicated as follows [30,31]


$$n_{o}\left(\Delta T,\omega_{Ge}\right)=n_{clad}\times [1+(1\times 10^{-3}+3\times 10^{-6}\Delta T)\times \omega_{Ge}]$$ (7)

$$\nu_{l}\left(\Delta T,\omega_{Ge}\right)=5944\times [1-(7.2\times 10^{-3}-4.7\times 10^{-5}\Delta T)\times \omega_{Ge}]$$ (8)

Where ΔT is the change of temperature, and the room temperature is 20 °C. $w_{Ge}$ is the doping concentration of GeO_2_ in the core.

## 3. Resluts and analysis

### 3.1. Mode distribution

**Fig 1:**
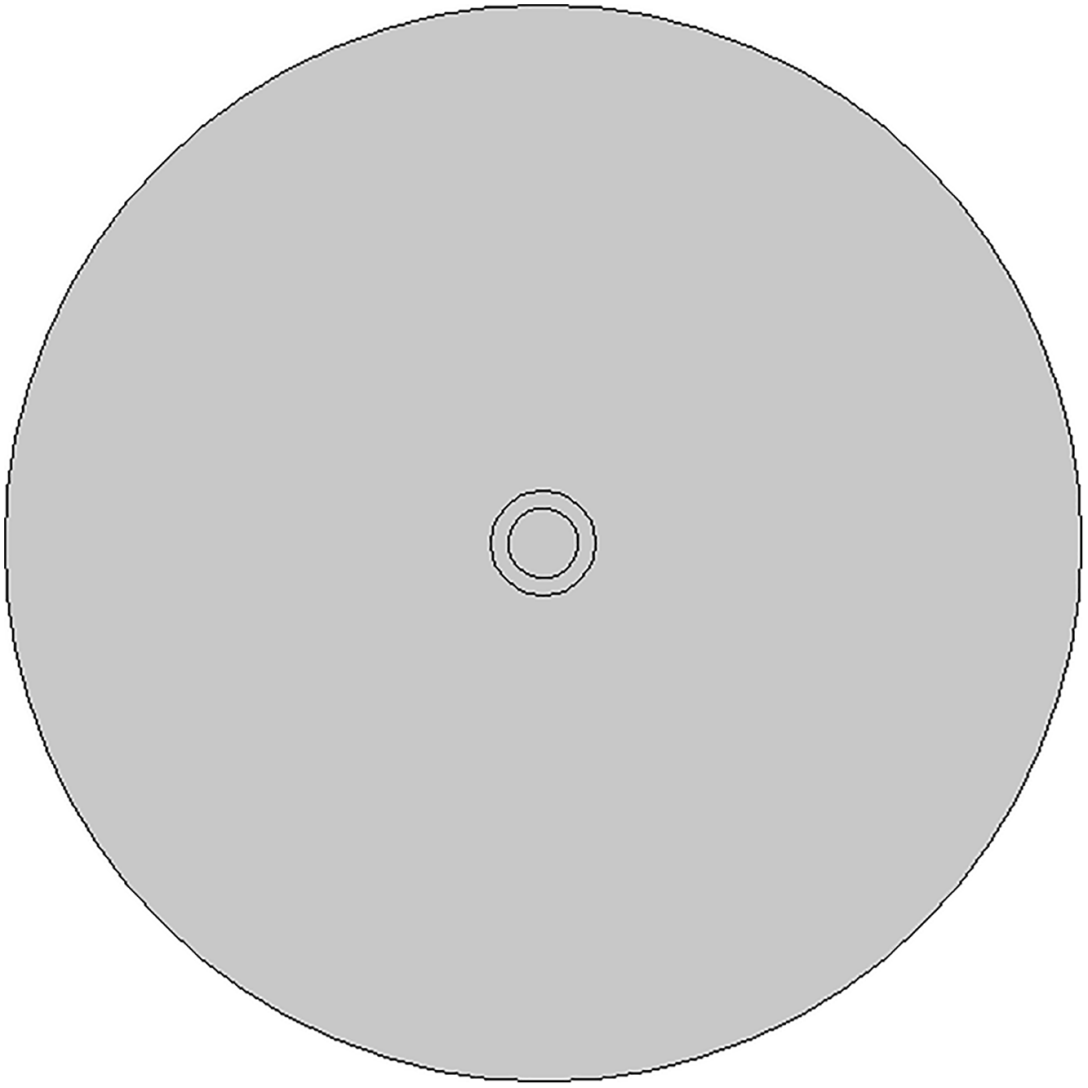
The structure of M-FMF.

A FMF with an M-shaped core refractive index profile is proposed for investigating the generation of SBS-based tunable slow light. Fig 1 illustrates the cross-sectional structure of the proposed M-FMF. The radius of the first and second core layers is 4 μm and 6 μm, respectively, with corresponding refractive indices of 1.454 and 1.4557 at 20 °C (at the wavelength of 1550 nm). The refractive index of the cladding is 1.444 under the same condition. Due to the fiber structure, only the LP_01_, LP_11_, LP_21_, and LP_02_ modes are supported in the proposed fiber and the other modes are cut off. In our simulation, only intramodal SBS and the changes caused by the peak value of the Brillouin main gain of each mode are taken into account.

**Fig 2:**
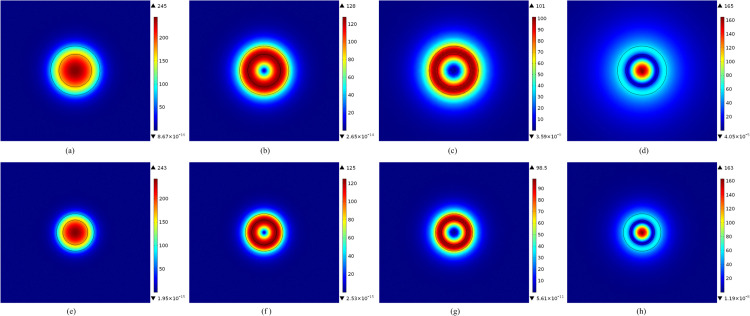
The distributions of the (a) LP_01_, (b) LP_11_, (c) LP_21_, and (d) LP_02_ optical modes at 20 °C, and the (e) LP_01_, (f) LP_11_, (g) LP_21_, and (h) LP_02_ optical modes at 80 °C in the M-FMF.

The electric-field distributions of the four optical modes in the M-FMF at temperatures of 20 °C and 80 °C are depicted in Fig 2. It can be seen that the light of the four optical modes is mainly concentrated in the core region. Moreover, at 80 °C, the optical field distributions of the four modes are significantly reduced compared to those at 20°C. This demonstrates that the confinement of light within the optical fiber core is enhanced. At identical temperatures, the fundamental optical mode LP_01_ depicted in Figs 2(a) and (e) exhibits the tightest confinement to the M-FMF core, followed by the LP_11_ mode (Figs 2(b) and (f)), and the LP_21_ mode (Figs 2(c) and (g)), while the LP_02_ mode (Figs 2(d) and (h)) demonstrates the weakest confinement.

### 3.2. Effective mode area

**Fig 3:**
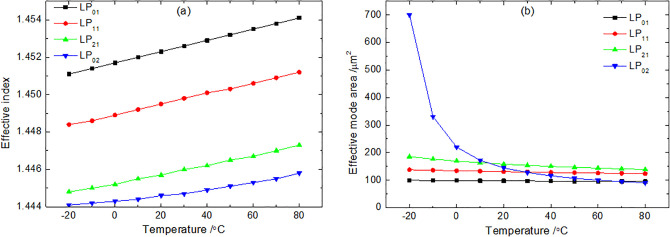
Variation of (a) the effective index and (b) the effective mode area with temperature.

The systematic analysis presented above reveals the fundamental thermo-optic governing principles behind the slow light performance in the proposed M-FMF. The core mechanism, as established in Eq. (7), is the temperature-induced increase in the material refractive index, which elevates both the core refractive index and the core-cladding index contrast. This directly leads to the observed increase in the effective refractive index $n_{eff}$ for all supported modes (LP_01_, LP_11_, LP_21_, and LP_02_) within the –20 °C to 80 °C range, as shown in Fig 3(a). Notably, at any given temperature, the effective refractive index $n_{eff}$ decreases in the order of LP_01_, LP_11_, LP_21_, and LP_02_. This hierarchy is intrinsically linked to the mode field distribution and confinement. A more critical consequence of this enhanced index contrast is the significant reduction in the effective mode area $A_{eff}$ with rising temperature, as evidenced in Fig 3(b). The strengthened fiber confinement compresses the optical field more tightly within the core. It is particularly noteworthy that the higher-order LP_02_ mode exhibits a dramatically larger variation in effective mode area (from 700.1 μm^2^ to 90.7 μm^2^) compared to the fundamental and lower-order modes. This distinct behavior stems from its field profile being more susceptible to changes in the waveguide boundary conditions imposed by the thermo-optic effect.

These parametric changes—increased $n_{eff}$ and decreased $A_{eff}$ —have a direct and profound impact on the SBS slow light characteristics. According to SBS theory, the Brillouin gain is inversely proportional to $A_{eff}$. Therefore, the substantial reduction in $A_{eff}$, especially for the LP_02_ mode, leads to a markedly enhanced SBS process. This is conclusively demonstrated in Fig 7 of Section 3.6, where the time delay and pulse broadening factor for the LP_02_ (and LP_21_) mode increase actively with temperature, while those for the LP_01_ and LP_11_ modes show a slight decrease. The LP_01_ mode maintains the largest time delay across the temperature range, indicating its SBS effect remains the strongest, consistent with its smallest $A_{eff}$ and highest $n_{eff}$.

### 3.3. Brillouin gain spectrum

**Fig 4:**
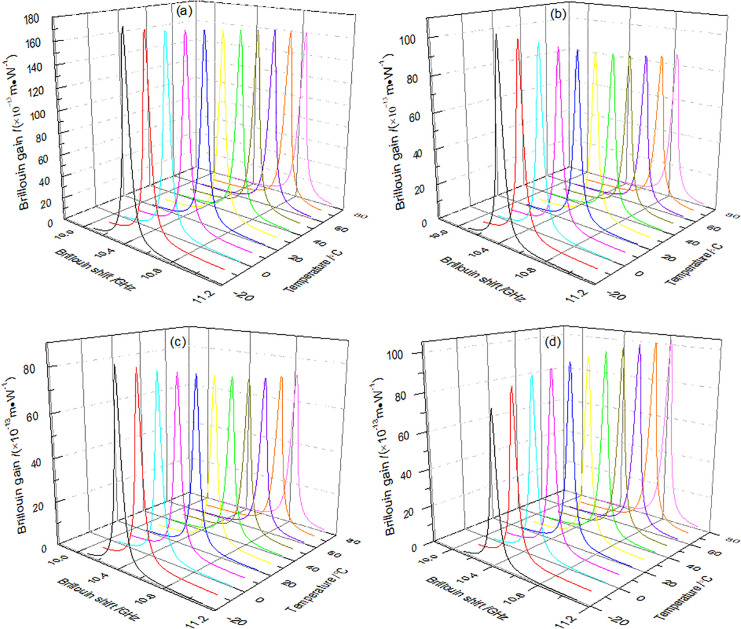
Brillouin gain spectra of the (a) LP_01_, (b) LP_11_, (c) LP_21_, and (d) LP_02_ modes in M-FMF.

Fig 4 shows the Brillouin gain spectra of different modes in the M-FMF. This illustration demonstrates the temperature-dependent Brillouin frequency shift and the concurrent appearance of the Brillouin gain peak. Notably, it is found that BFS values of the four modes increase within the temperature range from –20 °C to 80 °C. Table 1 lists the specific BFS values obtained under different temperatures. This behavior arises because the BFS depends on both the effective refractive index and the effective acoustic velocity of the mode. As the temperature increases, the effective acoustic velocity gradually rises, thereby leading to an increase in the BFS. This temperature-induced shift in the BFS directly modifies the Brillouin gain spectrum, as quantitatively shown in Fig 4. Consequently, the alteration of the gain spectrum profile changes the steep dispersion slope experienced by a probe signal tuned near the gain peak, which is the fundamental mechanism for generating slow light. Therefore, the change in BFS ultimately governs the temperature-dependent tunability of the slow light time delay. The simulated results of this direct impact—the variation of slow light time delay with temperature—are explicitly presented and analyzed in Fig 7(a) of Section 3.6.

**Table 1:** BFS values of the LP_01_, LP_11_, LP_21_, and LP_02_ modes at different temperatures.

Temperaure (°C) BFS(GHz)	−20	−10	0	10	20	30	40	50	60	70	80
LP_01_	10.437	10.476	10.515	10.554	10.593	10.632	10.671	10.710	10.749	10.788	10.827
LP_11_	10.431	10.469	10.509	10.548	10.588	10.627	10.666	10.705	10.744	10.784	10.823
LP_21_	10.420	10.460	10.499	10.539	10.578	10.618	10.657	10.696	10.735	10.775	10.815
LP_02_	10.418	10.457	10.496	10.535	10.574	10.613	10.652	10.691	10.730	10.769	10.809

The Brillouin gain peak of the LP_02_ mode increases with temperature, indicating that its SBS effect in the FMF is strengthened as temperature rises, in contrast, the LP_01_, LP_11_, and LP_21_ modes exhibit opposite temperature-dependent trends. Under identical conditions, among these three modes, the LP_01_ mode shows the highest Brillouin gain peak intensity, followed by LP_11_, with LP_21_ displaying the lowest value. When the temperature increases from –20 °C to 80 °C, the Brillouin gain peak of the LP_01_ (LP_11_, LP_21_) mode decreases from 1.812 × 10^-11^ m/W (1.068 × 10^-11^ m/W, 8.516 × 10^-12^ m/W) to 1.621 × 10^-11^ m/W (8.676 × 10^-12^ m/W, 7.356 × 10^-12^ m/W), that of the LP_11_ mode decreases from 1.068 × 10^-11^ m/W to 8.676 × 10^-12^ m/W, and that of LP_21_ mode decreases from 8.516 × 10^-12^ m/W to 7.356 × 10^-12^ m/W, while the LP_02_ mode increases from 7.90 × 10^-12^ m/W to 1.026 × 10^-11^ m/W. The average changes in Brillouin gain peak for the LP_01_, LP_11_, LP_21_, and LP_02_ modes are respectively 1.91 × 10^-12^ m/W, 2.004 × 10^-12^ m/W, 1.16 × 10^-12^ m/W, and 2.36 × 10^-12^ m/W, with the LP_02_ mode exhibits the largest variation. This indicates that the LP_02_ mode demonstrates greater sensitivity to temperature changes compared to other modes.

### 3.4. Overlap integral

**Fig 5:**
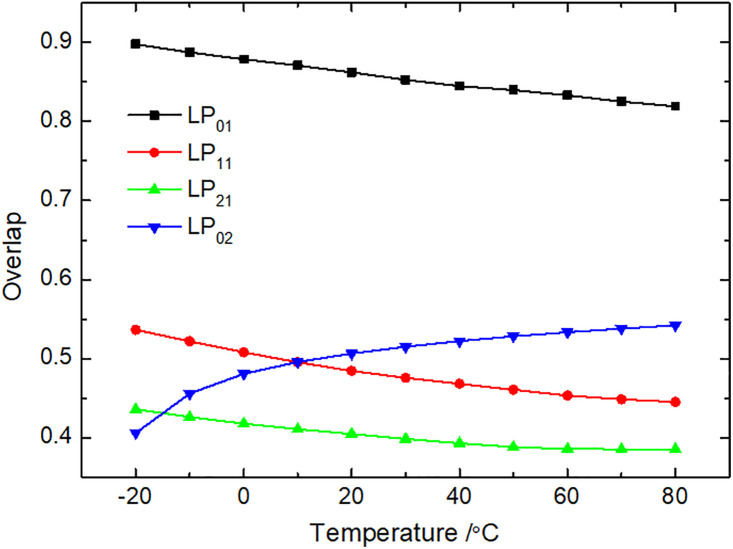
Overlap integrals of different modes in the M-FMF.

In the process of SBS in the M-FMF, the acousto-optic overlap integral is related to the coupled acoustic mode and optical mode. Fig 5 shows temperature-dependence curves of the acousto-optic overlap integral of different modes in the FMF. We observe that the overlap integrals of the LP_01_, LP_11_, and LP_21_ modes decrease with increasing temperature, which can be explained by the weakened ability of the fiber core to confine light, due to special structure of the M-type optical fiber. However, the LP_02_ mode exhibits opposite characteristic. The LP_02_ mode exhibits a markedly different temperature-dependent trend in coupling efficiency, SBS threshold, and slow light parameters compared to the LP_01_ and LP_11_ modes. This is attributed to its unique higher-order field structure, characterized by a ring-shaped intensity profile. This profile results in a distinct overlap with the acoustic phonon field and makes its effective mode area particularly sensitive to temperature-induced alterations in the M-FMF’s refractive index profile. Consequently, the SBS gain and the resulting nonlinear phase shift for the LP_02_ mode show a stronger and non-monotonic dependence on temperature, as reflected in its time delay and broadening factor. When the temperature increases from –20 °C to 80 °C, the coupling integral of the LP_01_ mode decreases from 0.897 to 0.819, that of the LP_11_ mode decreases from 0.536 to 0.445, and that of the LP_21_ mode decreases from 0.436 to 0.385, while the LP_02_ mode increases from 0.406 to 0.542.

### 3.5. Brillouin threshold

**Fig 6:**
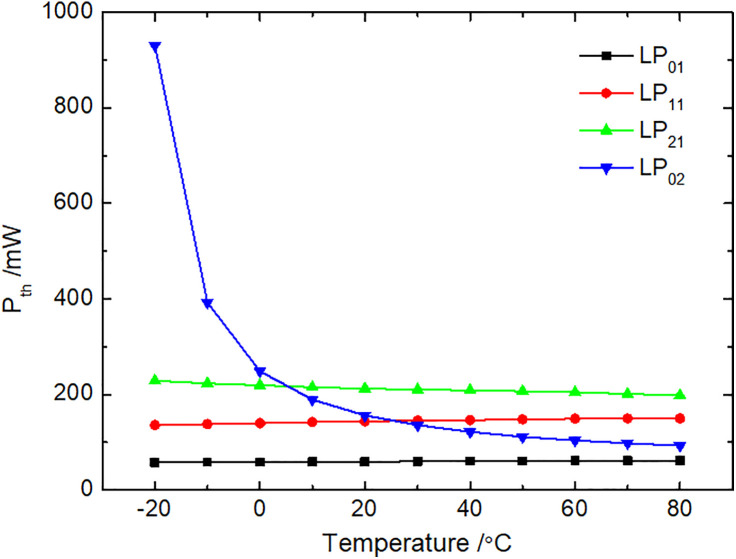
Variation of Brillouin threshold with temperature in the M-FMF.

The Brillouin properties of the spatial modes in the M-FMF differ slightly, and the simulation results based on Equation (3) are presented in Fig 6, showing the Brillouin threshold for each mode at varying temperatures. Fig 6 shows that the Brillouin threshold of the LP_02_ mode decreases with temperature, particularly exhibiting a significant drop between –20 °C and 20 °C. In contrast, the Brillouin thresholds of the LP_01_, LP_11_, and LP_21_ modes change minimally with temperature. The reduction in effective mode area and the enhancement of the SBS effect lead to a decrease in the Brillouin threshold required for SBS occurrence. Furthermore, over the entire temperature range of –20 °C to 80 °C, the LP_01_ mode has the smallest Brillouin threshold, indicating that it is the most prone to the SBS effect among the four modes. Specifically, within –20 °C to 5 °C, the LP_02_ mode has the largest Brillouin threshold, followed by the LP_21_ and LP_11_ modes. Within 5 °C to 80 °C, the LP_21_ mode exhibits the highest threshold. As the temperature increases from –20 °C to 80 °C, the Brillouin threshold of the LP_02_ mode decreases from 930.5 mW to 92.7 mW, and that of LP_21_ mode decreases from 228.6 mW to 198.3 mW, while the LP_01_ mode increases from 57.8 mW to 61.7 mW and the LP_11_ mode increases from 135.7 mW to 150.0 mW. The average variation of Brillouin threshold for the LP_02_ mode is the largest among the four modes, reaching 701.9 mW. This indicates that the LP_02_ mode is more sensitive to temperature variations, which is consistent with the conclusion in Section 3.3.

### 3.6. Time delay of slow light

**Fig 7:**
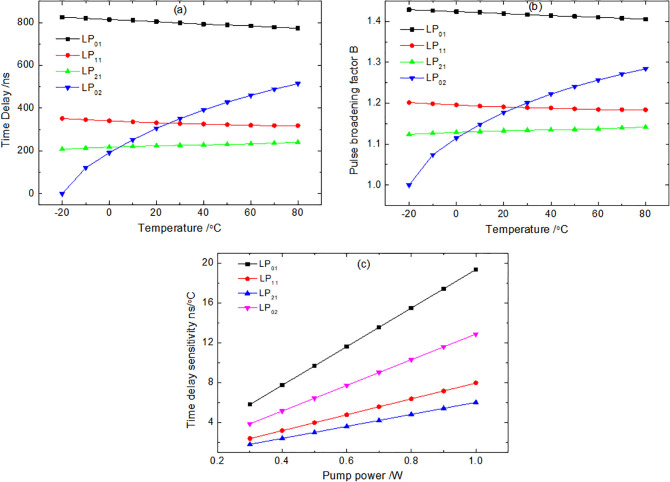
(a) Time delay, and (b) pulse broadening factor vary with temperature. (c) Time delay sensitivity varies with pump power.

The temperature-dependent slow light characteristics presented in Fig 7 reveal a distinct, mode-selective behavior within the M-FMF, which forms the core of our parametric study. From Fig 7(a-b), a key finding is the divergent response between mode groups: the time delay and pulse broadening factor for the higher-order LP_02_ (and LP_21_) modes increase significantly with temperature, whereas those for the fundamental LP_01_ and lower-order LP_11_ modes exhibit a moderate decrease. This fundamental divergence can be directly traced to the thermo-optic effect governing the fiber properties, as established earlier. The rising temperature increases the core-cladding refractive index contrast, which more strongly confines the optical field of higher-order modes (evidenced by their drastically reduced $A_{eff}$ in Fig 3(b)), thereby enhancing their SBS gain and consequent slow light effect. Conversely, the more stable confinement of the fundamental LP_01_ mode leads to its consistently dominant, yet slightly decreasing, SBS interaction across the temperature range.

This study provides a complementary perspective by quantitatively investigating the temperature-dependent evolution of key properties for the LP_02_ and other supported modes, and establishing how this evolution governs their SBS time delay—a direct performance metric for slow light-based sensors. The observed switch in the mode exhibiting the minimum time delay (from LP_02_ below 5°C to LP_21_ above 5°C) highlights the intricate and temperature-dependent relationship between modal confinement strength and SBS efficiency. The LP_02_ mode, with its pronounced temperature sensitivity (exhibiting a time delay change from 0 ns to 514.9 ns), is identified as a prime candidate for high-sensitivity temperature sensing. This marked response suggests the potential for achieving very high thermal resolution in sensor applications. Conversely, the relatively stable LP_01_ and LP_11_ modes (LP_01_ time delay change from 825.7 ns to 774.0 ns) are well-suited to act as stable reference channels in a multi-parameter sensor. Their low thermal sensitivity can be leveraged to compensate for common-mode noise or to measure other parameters, such as strain, with minimized thermal cross-sensitivity. Most importantly, the opposing temperature-dependent trends between the LP_01_/LP_11_ and LP_02_/LP_21_ mode groups inherently enable a differential or ratio metric sensing scheme. This approach can effectively isolate the temperature signal from other interfering perturbations, thereby enhancing measurement accuracy and enabling robust multi-parameter discrimination.

The sensitivity of the time delay to temperature (at 80 °C) for different pump power is shown in Fig 7(c). As can be seen, the time delay varies linearly with pump power. As mentioned above, compared with the LP_11_ and LP_21_ modes, the LP_02_ mode exhibits superior sensitivity. At a pump power of 0.3W, the sensitivities are 5.805 ns/°C for LP_01_, 2.388 ns/°C for LP_11_, 1.805 ns/°C for LP_21_, and 3.862 ns/°C for LP_02_. At 1 W, they increase to 19.350 ns/°C for LP_01_, 7.960 ns/°C for LP_11_, 6.018 ns/°C for LP_21_, 12.873 ns/°C for LP_02_, respectively. These results indicate that the time delay sensitivity can be effectively enhanced by increasing the pump power.

Within the framework of this foundational theoretical investigation, it is crucial to appropriately contextualize the presented performance metrics. Our analysis, based on numerical simulations under idealized conditions, establishes the theoretical upper bounds for intrinsic material and waveguide responses—such as the maximum achievable time delay (514.9 ns for the LP_02_ mode) and its intrinsic temperature sensitivity (12.873 ns/°C at 1 W of pump power). These parameters define the fundamental physical limits imposed by the M-FMF design; they are essential prerequisites for, yet distinct from, final system-level benchmarks. While indicators like the delay-bandwidth product (which is more pertinent to communication buffers than to the quasi-static sensing targeted here), signal-to-noise ratio, and ultimate sensing resolution are vital for evaluating a complete sensor system, their rigorous quantification inherently lies beyond the primary scope of this work. Such practical metrics are highly system-dependent, being influenced by experimental implementation details—including laser noise, detection electronics, and environmental shielding—as well as by challenges such as long-term stability and cross-sensitivity to strain or other perturbations. Consequently, a comprehensive evaluation of these practical benchmarks constitutes the logical and essential next step. This will be pursued through the construction and characterization of an experimental prototype based on the design principles elucidated here (e.g., utilizing the highly sensitive LP_02_ mode or a differential sensing scheme), with the primary goal of validating the practical potential of M-FMF-based slow-light temperature sensing.

### 3.7. Output wave

**Fig 8:**
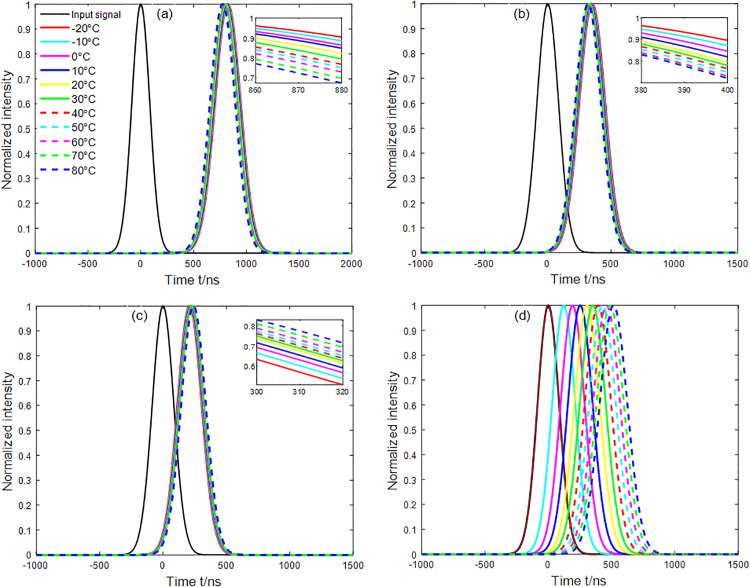
Variation of the Stokes wave with temperature in the M-FMF: (a) LP_01_ mode, (b) LP_11_ mode, (c) LP_21_ mode, and (d) LP_02_ mode.

The variations in the output signal waves for the four modes at a wavelength of 1550 nm as a function of temperature are depicted in Fig 8. In this figure, a noticeable time delay and broadening of the output signals are observed compared to the input signal wave, which has an input pulse width of 200 ns. As shown, the output waves of all four modes exhibit varying degrees of broadening, and the time delay varies significantly with temperature, particularly for the LP_02_ mode. This indicates that LP_02_ mode is more sensitive to temperature variations, which agrees with the conclusion in Section 3.6. Simulation results show that the pulse broadening factor increases with the time delay. Additionally, time delay is consistently associated with pulse distortion, implying that any increase in time delay degrades pulse quality due to greater distortion.

## 4. Conclusion

This study investigates temperature sensing characteristics based on slow light generation via SBS in the M-FMF. Through theoretical analysis and numerical simulations, we examine how temperature variations affect the effective mode area, Brillouin gain spectrum, overlap integral, and slow light time delay for four modes: LP_01_, LP_11_, LP_21_, and LP_02_. The results reveal that as the temperature increases, the slow light time delay of the LP_02_ mode increases significantly, along with an increase in the pulse broadening factor, indicating its high sensitivity to temperature. In contrast, the LP_01_ mode exhibits the largest time delay and the strongest slow light performance throughout the tested temperature range. This work provides foundational insights by establishing a comparative theoretical framework. It identifies the LP_02_ mode as the most promising candidate for sensing due to its superior overlap integral and thermo-optic response, thereby laying a necessary theoretical foundation for practical M-FMF temperature sensors. The logical next steps are to build an experimental prototype for validation and to develop more comprehensive models that incorporate dynamic effects, such as pump depletion and temperature-dependent acoustic damping, to enable precise system-level prediction.

## Supporting information

S1 Data Raw datasets. The Excel files contain the raw data analyzed in the manuscript.
(XLSX)
